# Ganglion of the Flexor Tendon Sheath at the A2 Pulley - Case Report

**DOI:** 10.5704/MOJ.1503.002

**Published:** 2015-03

**Authors:** P Gunaseelan, P Jeremy, CK Chua, F Rashdeen

**Affiliations:** Department of Orthopaedics, Universiti Kebangsaan Malaysia, Kuala Lumpur, Malaysia; * Hand and Microsurgery Unit, Hospital Kuala Lumpur, Kuala Lumpur, Malaysia

**Keywords:** Hand, Tendons, Ganglion cyst, A2 pulley

## Abstract

There are few reported cases of flexor tendon sheath ganglion arising from the A2 pulley. We report a case of a flexor tendon sheath ganglion in a 17-year old female who presented with pain, triggering and a swelling at the base of her right ring finger. During the excision biopsy, a ganglion measuring 0.5×0.8×0.4 cm in size was removed from the A2 pulley area.

## Introduction

Ganglions are commonly found in specific locations on the hand, wrist and feet. They are usually soft, solitary and asymptomatic, occasionally causing pain and neurologic symptoms if they are located adjacent to a peripheral nerve^1^. Ganglion is the most common soft tissue tumour of the hand, representing approximately 50% to 70% of all upper extremity swellings but the exact aetiology remains obscure.2 The female to male ratio of ganglions in the flexor tendon sheath has been reported as 2.6 to 1 and is predominant in females in their third and fourth decades of life. The A2 pulley is rarely involved.2 We report a case of a ganglion originating from the flexor tendon sheath at the A2 pulley.

## Case Report

**Fig. 1:**
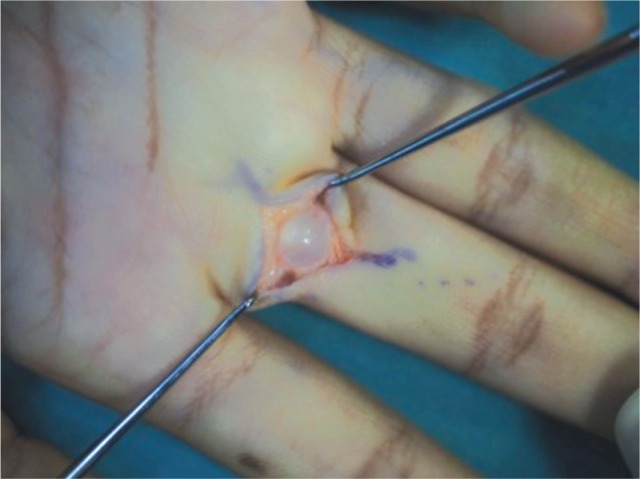
Ganglion in situ.

**Fig. 2:**
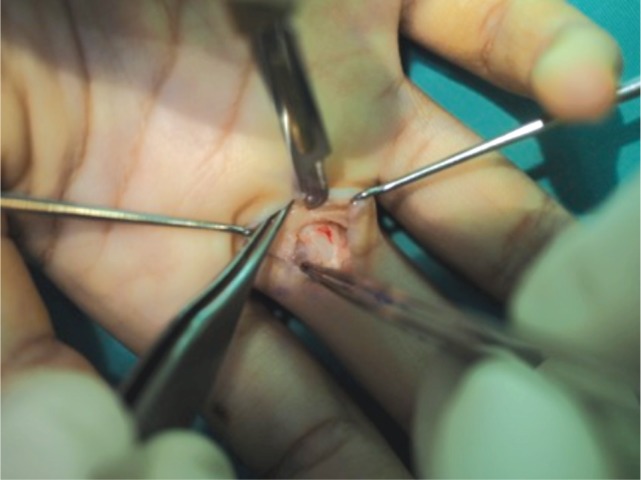
Ganglion excised with a portion of A2 pulley.

A 17-year old girl presented with pain on the palmar aspect of the metacarpo-phalangeal (MCP) joint of her right ring finger for the past few months. She was a pianist and had no significant past medical history. She complained of pain on finger flexion, and mild triggering was observed at the proximal interphalangeal joint (PIPJ). A small cystic mass was palpable in the subcutaneous layer over the proximal finger flexion crease. The mass was fixed to the underlying structures during flexion and extension of the affected digit. The excision of mass was done via a transverse incision parallel to the proximal finger flexion crease under loupe magnification. The mass was located on the proximal part of A2 pulley (Fig 1 & 2). No complications were noted at the last appointment, 6 months after surgery.

## Discussion

Ganglions have been recognized since the time of Hippocrates, who suggested that they represent mucoid flesh, thus differentiating them from ganglions of neural origin. Although much has been written about this common hand tumour, little attention has been given to flexor tendon sheath ganglions, which are often referred to as volar retinacular cysts or “sesamoid” or “pearl-seed”ganglia. This seems particularly surprising given that they are relatively common and often result in significant discomfort and loss of normal hand function.

The aetiology of the flexor tendon sheath ganglion is unknown^1^. Eller suggested that they originate from a localized rupture within the flexor tendon sheath^2^. Doyle believed that they result from a rent in the synovial membrane that heals but subsequently leaves an extracapsular piece of membrane that continues to secrete synovial fluid. Nelson *et al*. have suggested a traumatic aetiology, with a preceding injury occurring in approximately half of all patients^2^. However, the findings in our case are similar to others in that we could not find a clear association between antecedent trauma and the subsequent development of a flexor tendon sheath ganglion.

Involvement of the flexor tendon sheath occurs in approximately 5 to 16% of all hand and wrist ganglions^3^. As has been reported with ganglions located in other areas of the hand, flexor tendon sheath ganglions seem to occur more commonly in women. The greater prevalence of young women with flexor tendon sheath ganglions has been attributed to this population’s concern about cosmesis^3^. This seems unlikely, given that a flexor tendon sheath ganglion is usually quite small and inconspicuously located on the palmar surface of the hand^3^. In our patient’s age group, cosmesis was not a major concern, which has been similarly noted by others.

Our patient presented with the clinical complaints of a painful, tender mass. The mass was small and firm in nature. The resulting discomfort was particularly troublesome with grasping activities such as holding a steering wheel or lifting objects. She also reported that the pain interfered with her hand function. Flexor tendon sheath ganglions may be painful because of local neural innervation of the cyst, which has been demonstrated histologically by DeOrsay *et al*. Certainly, the level of disability in the patient with a ganglion of the flexor tendon sheath can be disproportionate to the size of the cyst. However, the resulting discomfort did not appear to cause our patient to urgently seek care considering that the symptoms were present for about nine months prior to presentation. This delayed presentation has also been noted by Matthews, who reported approximately 10 months of symptoms prior to presentation in his series. Our patient noted associated triggering of the affected digit. The association between stenosing tenosynovitis (trigger finger) due to ganglion at the A2 pulley has not been previously reported^4^.

A variety of treatment modalities had been advocated for ganglions in general. Manual compression was the simplest yet least effective treatment approach^5^. Theoretically, it should not be as successful for flexor tendon sheath ganglions because of their small size and tenderness. Radiation therapy was advocated in the early 1940s, but this modality was no longer utilized because of concerns regarding the long-term sequelae of radiating the hand^5^. Transfixion with a loop of catgut suture had also been advocated, with a reported failure rate of only 1%. Aspiration of the cyst followed by injection with sclerosing agents such as iodine, carbolic acid, or ethanolamine had also been utilized but no longer advocated because of the risk of soft tissue necrosis^5^. Currently, the most popular treatment method involves aspiration with steroid injection but the risk of recurrence is high. Excision has been advocated because of the potential for digital nerve injury and the high recurrence rates reported with aspiration^5^. However, the results of surgical excision of a flexor tendon sheath ganglion are difficult to interpret because most series have grouped them with other hand and wrist ganglions. There are only two studies that have specifically assessed the results of excision of flexor tendon sheath ganglion. Bittner **et al**. reported a recurrence in only one of their 43 patients who underwent cyst excision^5^. Resection with a surrounding portion of the flexor tendon sheath is recommended to minimize the chance of recurrence because it has been suggested that recurrence is the result of inadequate removal of all degenerative myxoid tissue. We have confirmed that surgical excision of a small part of the distal aspect of A2 pulley does not cause any bowstring of the flexor tendon and results in a low recurrence rate, minimal complications, and high patient satisfaction.
